# Novel CAD/CAM-splint-based navigation protocol enhances intraoperative maxillary position control in orthognathic surgery: a case control study

**DOI:** 10.1186/s13005-024-00477-3

**Published:** 2025-01-10

**Authors:** Felix Schrader, Leonardo Saigo, Norbert Kübler, Majeed Rana, Max Wilkat

**Affiliations:** 1 Department of Oral and Maxillofacial Surgery, Heinrich Heine University Hospital Düsseldorf, Moorenstraße 5, 40225 Düsseldorf, Germany; 2 Department of Oral and Maxillofacial Surgery, National Dental Centre Singapore, 5 Second Hospital Ave, Singapore, 168938 Singapore

**Keywords:** Surgical navigation, Orthognathic surgery, Computer-assisted planning, Intra-oral scanning, Navigational registration, CAD/CAM splint

## Abstract

**Background:**

Virtual surgical planning for orthognathic surgery typically relies on two methods for intraoperative plan transfer: CAD/CAM occlusal splints and patient-specific implants (PSI). While CAD/CAM splints may offer limited accuracy, particularly in the vertical dimension, PSIs are constrained by higher costs and extended preparation times. Surgical navigation has emerged as a potential alternative, but existing protocols often involve invasive registration or lack transparent evaluation. This study introduces a novel protocol for point-based optical navigation using modified CAD/CAM splints for non-invasive registration and transparent intraoperative evaluation, assessing its effectiveness in maxillary positioning.

**Methods:**

This prospective case-control study included 20 patients undergoing bimaxillary orthognathic surgery. The experimental group employed surgical navigation with modified CAD/CAM splints, while the control group used standard CAD/CAM splints. Surgical accuracy was evaluated by measuring translational and rotational discrepancies between the planned and achieved maxillary positions. A mixed ANOVA was conducted to assess other factors, aside from surgical navigation, that might influence surgical accuracy.

**Results:**

Surgical navigation significantly improved accuracy in translational movements along the x-axis (right-left: -0.81 mm; *p* = 0.021) and z-axis (down-up: -0.82 mm; *p* = 0.014), as well as in yaw rotation (-0.45°; *p* = 0.045). Other movements also showed improved precision in the navigated group, though not statistically significant; y-axis (back-front): -0.60 mm (*p* = 0.094); pitch rotation: -0.70° (*p* = 0.071); roll rotation: -0.04° (*p* = 0.428). Besides the use of surgical navigation, the amount of planned movement significantly impacted surgical accuracy, although no specific factors could be identified to predict which cases would particularly benefit from surgical navigation.

**Conclusions:**

Surgical navigation with modified CAD/CAM splints enhances surgical accuracy without requiring invasive procedures, offering a straightforward and transparent protocol suitable for routine clinical practice that allows intraoperative evaluation of maxillary positioning. However, the clinical significance and cost-effectiveness compared to PSI need further investigation. These findings suggest new directions for future developments, especially with advancements in mixed reality technologies, which could broaden the application of surgical navigation.

**Trial registration:**

Retrospectively registered with the German Clinical Trials Register (DRKS00034795).

**Supplementary Information:**

The online version contains supplementary material available at 10.1186/s13005-024-00477-3.

## Introduction

Orthognathic surgery remains a crucial intervention in addressing skeletal discrepancies within the maxillo-mandibular complex, effectively treating malocclusion and enhancing facial aesthetics [1, 2]. The collaborative approach of orthodontic treatment coupled with surgical correction becomes imperative in rectifying these anomalies, often initiated after pre-surgical orthodontic preparation [3].

The significance of meticulous procedure planning has been integral to orthognathic surgery from its inception [4]. Recently, virtual surgical planning has become synchronous with orthognathic surgery, offering improved simulation and heightened aesthetic outcomes [5–7]. Despite the advantages of virtual planning, the translation of a plan into reality requires careful consideration, typically involving two options: 3D-printed CAD/CAM-occlusal splints or patient-specific implants (PSI) [8, 9].

While CAD/CAM-occlusal splints present limitations, especially in vertical accuracy referencing the mobile mandible rather than the skull base, PSI has been deemed the gold standard for its high accuracy in all dimensions [10, 11]. However, the downsides of PSI including high cost, extended preparation time and limited adaptability during surgery can pose challenges and have been discussed in literature [11–13].

Surgical navigation offers the possibility for an intra-operative, real-time evaluation of surgical accuracy and poses therefore a third option of transferring a plan to reality [14, 15]. While it is widely established in craniomaxillofacial surgery for certain indications like traumatology and oncology, it has not been utilized for orthognathic procedures as a standard tool [16–20]. With emerging technologies like mixed reality or surgical site projection which fundamentally rely on the basic principles of surgical navigation, its applicability is further being expanded, overlaying the real world with virtual DICOM-based planning data [21–23]. However, a successful navigation procedure hinges on three critical aspects: tracking the patient in real-time, accurate registration and meticulous evaluation of 3D object positioning, a challenge given the six degrees of freedom for movement [24, 25].

While promising results have been reported in literature regarding the use of surgical navigation for orthognathic surgery, procedural details are often lacking, limiting repeatability and transparency in results [26]. Furthermore, reported procedures frequently involve invasive or inaccurate registration, and in-transparent intra-operative evaluation. This paper introduces a novel protocol utilizing CAD/CAM splints modified for surgical navigation, offering non-invasive registration and facilitating a transparent and accurate intra-operative evaluation.

## Methods

This clinical prospective case control study was conducted according to the Declaration of Helsinki. It was approved by the Ethics Committee of the Heinrich Heine University Düsseldorf (study number: 2023–2716; date of approval: April 29, 2024). The study was retrospectively registered in the German Clinical Trials Register of the Federal Institute of Drugs and Medical Devices (registration number: DRKS00034795; date of registration: July 31, 2024). All patients provided their consent to participate in the study and for the use of their data.

### Planning procedure

**Fig. 1:**
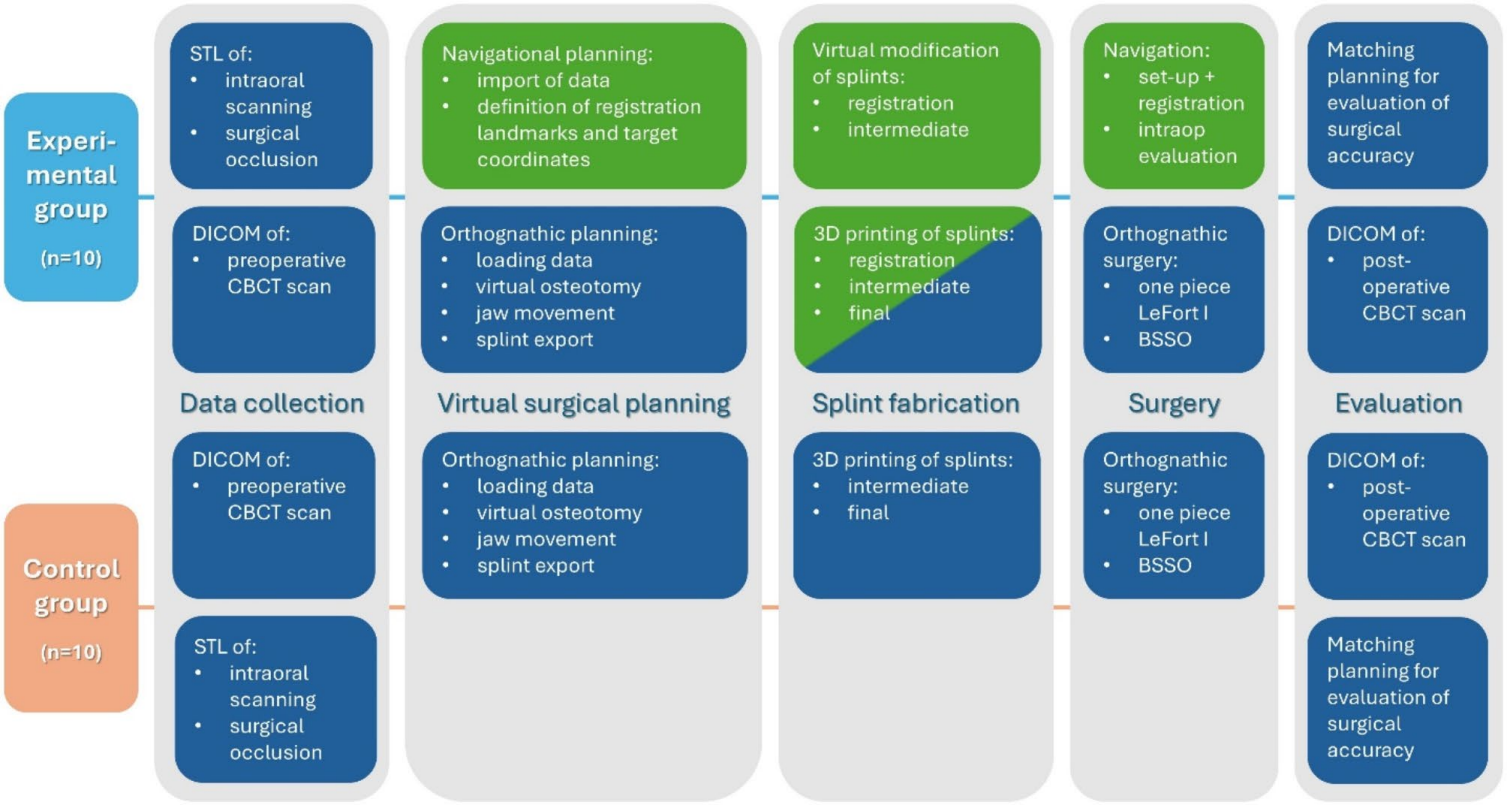
Study Protocol The surgical planning, treatment and evaluation workflow steps for both the experimental and control groups are depicted. Steps common to both groups are shown in dark blue, while additional steps related to the execution of surgical navigation for the experimental group are highlighted in green

A standardized protocol was established based on CAD/CAM splints with the integration of design features allowing the usage of surgical navigation for orthognathic bimaxillary surgery (see Fig. 1).

#### Virtual surgical planning

For virtual surgical planning, data collection comprised a cone beam computed tomography (CBCT) scan capturing the patient’s facial skull in the initial occlusion with a central fossa-condyle relationship using a wax bite. Additionally, intraoral scans of both upper and lower dentitions were taken, along with a third surface scan merging both dentitions in the final surgical occlusion relationship using 3D-printed dental resin casts. Utilizing the IPS Case Designer software (V2.3.5.2, KLS Martin, Tuttlingen, Germany), virtual surgical planning involved alignment of the DICOM data according to the Frankfurt horizontal plane and midfacial sagittal plane followed by semi-automatic segmenting of the facial skull. The user approved the threshold for hard and soft tissue. Subsequently, the two intraoral scans of the upper and lower dentition were loaded in STL format and automatically matched to the DICOM dataset. This step yielded a virtual reconstruction of the facial skull, integrating high-resolution geometry information from the intraoral scan at the occlusion surfaces of the teeth.

The subsequent stage encompassed virtual outlining of osteotomy lines of Le Fort I osteotomy and bilateral sagittal split osteotomies (BSSO), along with planning jaw movements. Typically, this included translational and rotational movements of the upper jaw, with the distal segment of the lower jaw aligning to the loaded surgical occlusion. Once planning was concluded, the software facilitated the design of occlusal splints for intermediate and final positions per the maxilla-first protocol, exportable in STL file format. Further structures such as the maxilla in the original position and the final planned position were exported in STL file format.

**Fig. 2:**
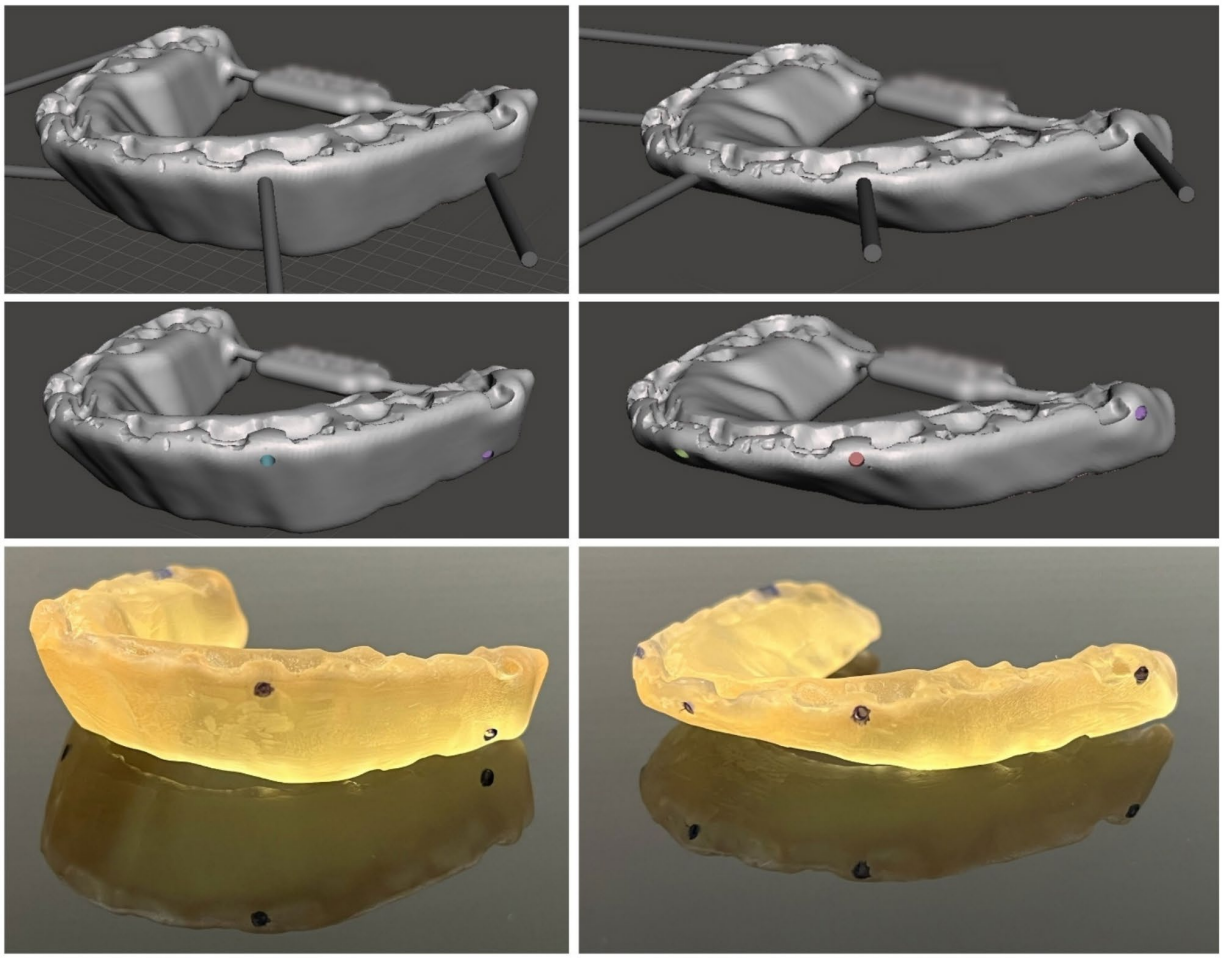
CAD/CAM fabrication of splints. The virtual modification of the registration (left) and intermediate splints (right) was performed using Autodesk Meshmixer freeware. Boolean subtraction of a cylindrical shape created indentations of the required depth and diameter. Four indentations were applied to the registration splint, distributed along its vertical axis, while five indentations were added to the intermediate splint, positioned at the first upper molars, canine tips and incisal point. Additive 3D-printing then converted the virtual plan into surgical resin splints, with indentations marked manually with a pen

For surgical navigation, a third splint was generated which served as the registration splint (see Fig. 2). This splint aligned with the intermediate splint of the mandible-first protocol featuring a wide-open locked bite with at least 10° autorotation of the mandible around the intercondyle axis. This resulted in a splint with a substantial vertical height, aligning with the original position of the maxilla as a prerequisite to serve as the registration splint. A detailed description of the design of the registration splint along with an evaluation of this non-invasive registration method has been published previously [27].

#### Splint modification and fabrication

Two of the three exported STL file format splints underwent digital modifications (see Fig. 2). Utilizing Autodesk Meshmixer freeware (Autodesk Research, San Francisco, USA), circular indentations with a diameter of 1.5 mm and a depth of approximately 3 mm were created on the vestibular face of the splints by performing boolean subtraction between the splint and a cylindrical object of the mentioned diameter. These indentations facilitated precise intra-operative positioning of the Brainlab surgical navigation system’s probe (Brainlab AG, Munich, Germany), featuring a pointed end with a diameter of approximately 1 mm.

For the registration splint, four indentations were strategically placed at the region of the first upper molars left and right, and the canine teeth left and right, equally distributed along the vertical axis (two above and two beneath the occlusion plane). This distribution of indentations is necessary to improve accuracy of surgical navigation as a skew quadrilateral is put up in space between the 4 indentations covering the area of interest for the navigated procedure [27].

The intermediate splint had five symmetrically distributed indentations across the upper dental arch within the occlusion plane (upper first molar left and right, upper canine left and right, upper incisal point).

The two modified splints as well as the unmodified final splint were imported into the Preform software (version 3.27.1, Formlabs Inc., Somerville, USA) for additive 3D printing preparation. Support structures were added without obstructing occlusal surfaces or navigational indentations. Utilizing surgical guide resin V1 in the 3D-printer Form 2 (Formlabs, version 3.27.1, Formlabs Inc., Somerville, USA) and a slice thickness of 0.05 mm, the 3D printing process was executed. After completing 3D printing and post-processing as per the manufacturer’s protocol, navigational indentations were marked using a pen (see Fig. 2).

#### Navigational planning

**Fig. 3:**
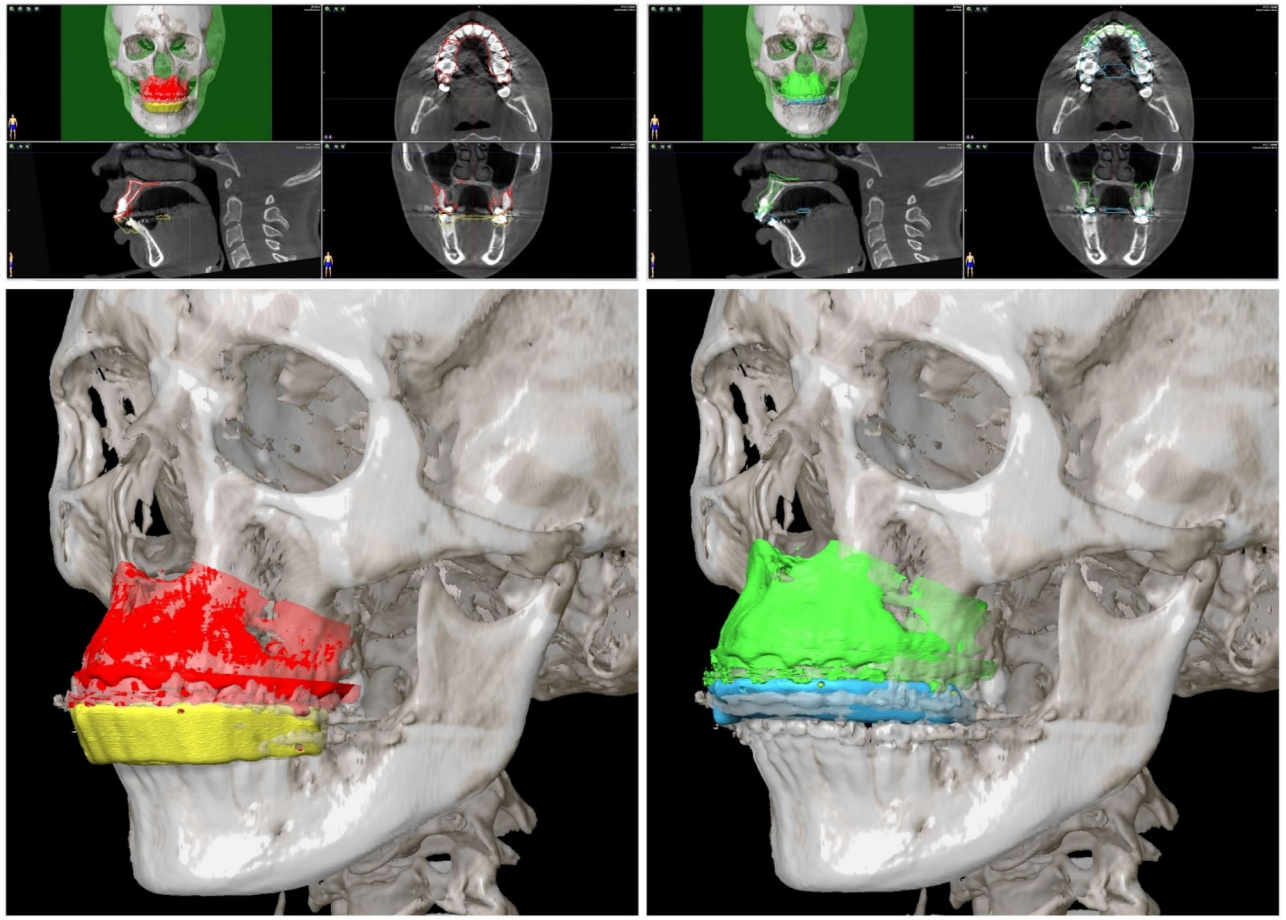
Navigational planning. The dataset was aligned to the Frankfurt horizontal and mid-sagittal plane. The loaded STL file of the maxilla in its original position (red) accurately aligned with the DICOM data and the registration splint (yellow). Registration landmarks were marked at the four indentations of the registration splint (red spheres). The planned maxilla position (green) aligned with the intermediate splint (blue), and target coordinates were marked at the five indentations of the intermediate splint (green spheres)

For preparation of surgical navigation, the DICOM data of the same preoperative CBCT scan which was used for the orthognathic planning was imported into iPlan CMF 3.0.5 (Brainlab AG, Munich, Germany). Data set was aligned according to the Frankfurt horizontal plane and midfacial sagittal plane (see Fig. 3). Before importing the exported STL files of the splints and the maxillas, these files had to be rotated by 180° around the z-axis to align to the world axis of the Brainlab system. Rotation of STL files was performed using Geomagic Freeform (version 2020.1.1, Oqton, Los Angeles, USA). The rotated STL files were imported into the iPlan CMF software. Four registration points were set on the surface of the bottom of the four indentations on the registration splint (see Fig. 3). Target coordinates of the planned final position for the five dental landmarks (upper first molar left and right, upper canine left and right, upper incisal point) were marked on the surface of the bottom of the five indentations on the intermediate splint (see Fig. 3).

### Orthognathic surgery with navigational control of maxillary positioning

Bimaxillary orthognathic procedure was performed with the help of surgical navigation (experimental group) utilizing the demonstrated protocol with modified CAD/CAM dental registration splint and CAD/CAM intermediate splint for intra-operative evaluation of maxillary positioning. A control group undergoing surgery with CAD/CAM splints without modification was utilized for comparative evaluation.

#### Patient selection

A continuous case series of twenty eligible patients who met the inclusion criteria were included in the study. While the first ten patients were operated on with the assistance of surgical navigation according to the presented protocol, the last ten patients underwent the same surgical procedure without the utilization of surgical navigation. All surgeries were performed by the same surgical team. Inclusion criteria were as follows:

- Patients in need of primary treatment for bimaxillary surgery presenting to the Department for Oral & Plastic Maxillofacial Surgery at the Heinrich Heine University Hospital Düsseldorf between 04/2024 and 08/2024.
- Patients aged 18 years and above at the time of surgery, having legally consented to participation in this prospective study.
- Maxilla-first surgical protocol
- Presence of complete data sets: DICOM data of the pre- and post-surgical cone-beam computed tomography (CBCT) scan encompassing the entire facial skull, intraoral scans of upper and lower dentition, surface scan of final surgical occlusion.

Exclusion criteria were as follows:

- Congenital craniofacial deformity or lip, jaw, palate clefts in the history
- Obstructive sleep apnea as indication for surgery
- Multi-segmental maxilla
- Additional surgical measures besides bimaxillary osteotomy (genioplasty, removal of third molars)

#### Surgical procedure

**Fig. 4:**
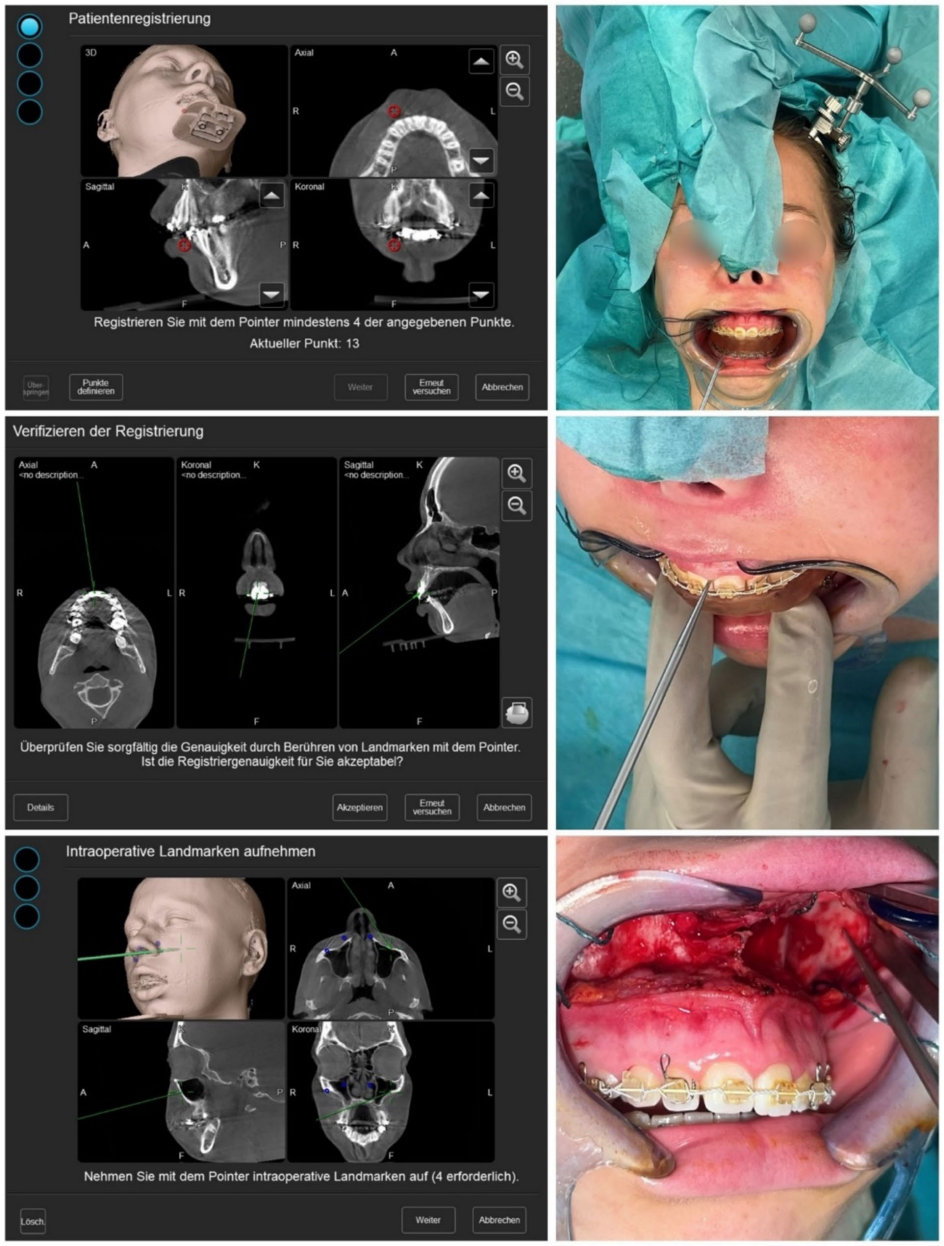
Registration process. The skull reference array was fixated and registration splint fitted on the upper dentition, registration process was completed by pointing at the four indentations of the registration splint with the navigation pointer (upper row). The accuracy of registration was verified using anatomical landmarks, such as the interproximal space of the central incisors (middle row). Four burr holes above the planned LeFort I osteotomy line were acquired as “rescue points” using the “acquire” option in “registration” mode (lower row)

Surgery was performed under general anesthesia with nasal intubation. Surgical navigation was performed using optical navigation system Curve (Brainlab AG, Munich, Germany). A small incision was made behind the hairline to attach the skull reference array at the left anteroparietal bone. The array was fixated using an 8 mm x 1.5 mm screw (KLS Martin, Tuttlingen, Germany). The registration splint was then placed on the upper dentition. Patient-to-image registration was done by positioning the navigation probe at the pre-determined indentations on the registration splint, aligning them to the same indentations pre-marked in the CBCT images (see Fig. 4). The result of registration could be verified by placing the probe on identifiable anatomical landmarks such as the incisal point or molar cusp tips (see Fig. 4).

After exposing the maxilla via a vestibular incision, the osteotomy lines were outlined with a pen. Subsequently, vertical reference points were made above and below the osteotomy lines with a 1 mm diameter round bur in the vertical axis, with a 10 mm spacing. Two sets of reference points were created on either side of the maxilla. The superior reference points located above the osteotomy line were targeted with the navigation probe and marked in the system using the “acquire” function in “registration” mode (see Fig. 4). These points served as “rescue points” for re-registration when necessary after mobilization of the maxilla, similar as reported by Shirota et al. [28].

**Fig. 5:**
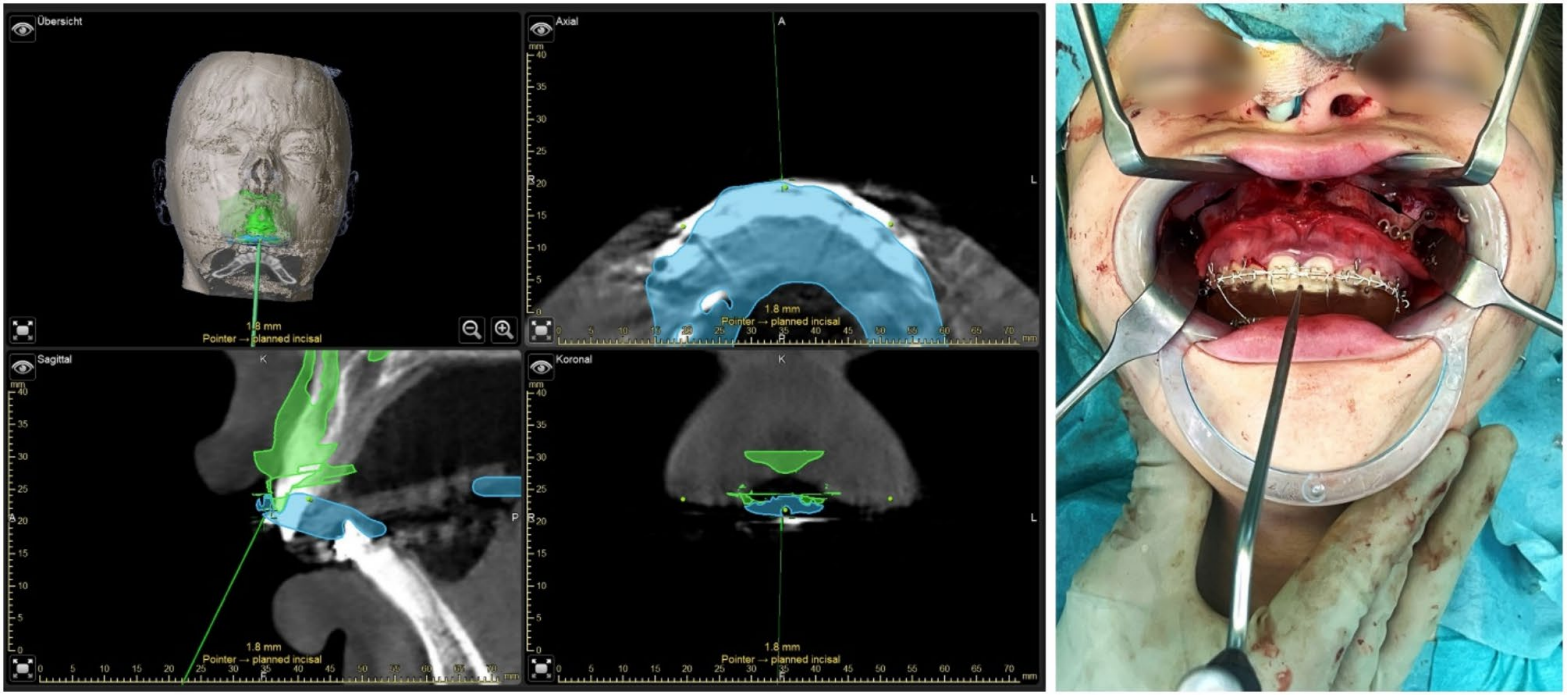
Intra-operative evaluation of maxillary positioning using surgical navigation. The maxillo-mandibular complex was fixed using the intermediate splint and wires, allowing autorotation until bony contact. Maxillary positioning was evaluated with the navigation probe at all five indentations of the intermediate splint. The Euclidean distance between the probe and target coordinate was displayed alongside a multiplanar view, guiding axis adjustments through bone trimming. This process could be repeated as needed, even after partial or complete osteosynthesis

The osteotomy was then performed using sagittal saw and completed using osteotomes along the maxillary buttresses, allowing downfracture and mobilization of the maxilla. The intermediate splint was then placed, and temporary maxillo-mandibular fixation was performed with wires. The maxillo-mandibular complex was autorotated, and the repositioning was evaluated using surgical navigation by pointing at the indentations of the intermediate splint (see Fig. 5). The navigation display showed the euclidean distance *d*, which can be defined through the equation.

$$d = \sqrt {{{({x_p} - {x_i})}^2} + {{({y_p} - {y_i})}^2} + {{({z_p} - {z_i})}^2}}$$

with the intraoperatively acquired real-time coordinates (x_i_, y_i_, z_i_) of the pointed indentation of the intermediate splint and the planned coordinates (x_p_, y_p_, z_p_) of the corresponding targets according to the planning dataset. To achieve a surgical precision of less than 1 mm for translational movements and less than 2° for rotational movements along the three spatial axes, a threshold of 1.7 mm for the displayed Euclidean distance was established according to the above formula. This threshold applies to both the incisal point (corresponding to translation since the center of planned rotation is located here) and the molars (corresponding to rotation) in an average dental arch. Considering a previously experimentally measured target registration error at the occlusion plane for the utilized registration method of approximately 0.8 mm [27], a total value of 2.5 mm was considered the upper limit during intraoperative evaluation, with the goal being to minimize the Euclidean distance value to as close to zero as possible.

In the multi-planar view, the axis with the greatest discrepancy could be visually identified and precisely measured using the digital ruler located at the edge of the image (see Fig. 5). Following analysis, further adjustments were made in the area of the osteotomy by trimming interfering bone to approximate the target position, and re-evaluation was performed. When an Euclidean distance close to zero or at least less than 2.5 mm at all 5 target coordinates was achieved, the maxilla was fixed with plates at the four buttresses. The navigation probe was used to confirm that the Euclidean distance matched the desired value at all five target coordinates. Surgery proceeded with BSSO and repositioning of the tooth-bearing mandibular segment using the final splint. Fixation of the manually positioned condyles was performed with plates. Once the adjusted bite was comfortably achieved, surgery concluded with closure of incisions.

In the control group, surgery was performed without usage of the navigation system. Intermediate and final CAD/CAM splints without indentations were used following the standard maxilla-first protocol. Vertical repositioning of the maxilla was guided by linear measurements at the intra-operative vertical reference points, as described previously.

#### Postoperative evaluation

Postoperatively, a routine follow-up cone-beam computed tomography (CBCT) scan of the patients was performed within 3 days post-surgery. This CBCT scan was compared to the original planning file for both groups using the comparison function of the IPS Case Designer (V2.3.5.2, KLS Martin, Tuttlingen, Germany). The loaded post-surgical DICOM dataset was matched to align with the preoperative planning file at the region of the cranial base following the software‘s voxel-based matching algorithm. Subsequently, the deviations of the translational and rotational movements of the maxilla were compared between the planned position and the post-surgical scan and calculated as the difference along all three spatial axes in mm or degree, respectively.

### Statistics

Data collection and storage was carried out using Excel spreadsheets (Excel 14.0, Microsoft Corporation, Washington, USA). The statistical evaluations were carried out with the software IBM SPSS Statistics (IBM Corp. Released 2023. IBM SPSS Statistics for Macintosh, Version 29.0.1. Armonk. NY: IBM Coro).

Values for the primary outcome parameter surgical accuracy defined as the difference between the virtually planned and the surgically achieved position of the maxilla separated in translational and rotational movements along the three spatial axes were calculated and depicted in a boxplot.

Moreover, the absolute values of the mentioned surgical accuracy were calculated and used for further analysis. The Shapiro-Wilk test was performed to assess the normality of the data distribution. Comparisons between the experimental and control groups were evaluated using an independent two-sample one-tailed Student’s t-test, assuming homogeneity of variance, which was tested using the Levene’s and Brown-Forsythe tests. In case of lack of homogeneity of variance, Welch’s t-test was used. P-values of *p* < 0.05 were considered significant. A post-hoc power analysis was conducted using the software G*Power (version 3.1.9.6, University Kiel/Düsseldorf, Germany) [29, 30].

To evaluate whether specific cases particularly benefit from the use of surgical navigation, the overall root mean square (RMS) of the primary outcome parameter surgical accuracy, as defined above, was calculated, accounting for all translational and rotational movements. A multi-factor ANOVA was conducted to compare the overall RMS of surgical accuracy with pre-existing anomalies across all three spatial dimensions, including sagittal discrepancies according to Angle classification, vertical discrepancies in the presence or absence of an open bite, and lateral discrepancies in the presence or absence of facial scoliosis. Additionally, the RMS of surgical accuracy was compared to the overall RMS of the planned movement to determine whether cases with larger movement distances across the axes would benefit from the use of surgical navigation. The Shapiro-Wilk test was performed to assess the normality of the data distribution. Homogeneity of variance was tested using the Levene’s and Brown-Forsythe tests. P-values of *p* < 0.05 were considered significant. In the case of significant results, Bonferroni post-hoc analysis was conducted with a corrected level of significance. A post-hoc power analysis was conducted using the software G*Power (version 3.1.9.6, University Kiel/Düsseldorf, Germany) [29, 30].

## Results

### Patient population

Descriptive data of both study groups are depicted in Table 1, which show a comparable composition of both the experimental and the control group.

**Table 1:** Descriptive data of patient population

Group	Subjects	Age in years (mean ± SD)	Gender (F/M)	Angle class (I/II/III)	Open bite (Y/*N*)	Face asymmetry (Y/*N*)
Experimental (navigated)	10	23.1 ± 3.2	5/5	2/4/4	4/6	5/5
Control (non-navigated)	10	28.2 ± 8.1	7/3	2/4/4	4/6	4/6

SD: standard deviation; F: female; M: male; Y: yes; N: no

### Pre-operative planning

Pre-operative planning for orthognathic surgery provides all necessary data for surgical navigation following the presented protocol. As a result, the decision to use navigation can be made spontaneously during virtual surgical planning, particularly when complex jaw movements are identified. No additional data collection is required, making the protocol highly applicable and efficient in clinical settings without affecting patient scheduling, increasing the surgical team’s workload or exposing the patient to additional radiation.

Preparation time for this navigation protocol is limited to virtual splint modification and the import of data into the navigation software, taking approximately 15 min more than standard orthognathic planning (compare Fig. 1). If a navigation system is available, additional costs are minimal, covering only 3D-printing of one extra splint (registration splint) and sterilization of navigation instruments, making them negligible.

### Intraoperative handling

The navigation setup was straightforward, utilizing a simple and precise registration protocol. The mean fiducial registration error was 0.12 ± 0.1 mm across all four fiducials on the registration splint in all 10 navigated patients. Acquiring the four burr holes cranial to the LeFort I osteotomy lines as „rescue registration landmarks“ after the initial registration allowed for re-registration, which was necessary in 1 out of 10 cases when the registration splint could no longer serve as a reference due to the mobilized maxilla.

The integration of targets into the intermediate splint provided clearly defined target coordinates, enabling reproducible verification, while the use of intermaxillary dental splints facilitated accurate maxillary positioning without the need of technically demanding freehand control. Consequently, navigation was feasible in the operating room and largely followed standard splint-based orthognathic surgery procedures. However, point-based navigation does not track objects in all six degrees of freedom (DoF), requiring interpolation from the 5 defined target coordinates via assessment of the multiplanar views rather than direct monitoring. Despite this limitation, point-based discrepancies, especially for vertical adjustments, were valuable intraoperatively as they indicated at which site/region additional bony trimming was needed to reduce discrepancies. This approach parallels conventional model surgery planning, where information transfer is also point-based rather than 6 DoF object-based. Once hand-eye coordination is mastered, retrieving information from surgical navigation becomes intuitive for intraoperative use.

Analysis of surgical duration showed that the experimental group (257.8 ± 49.1 min) had an average surgery time approximately 30 min longer than the control group (227.7 ± 67.1 min), though this difference was not statistically significant (*p* = 0.292).

### Post-surgical accuracy of maxillary positioning

**Fig. 6:**
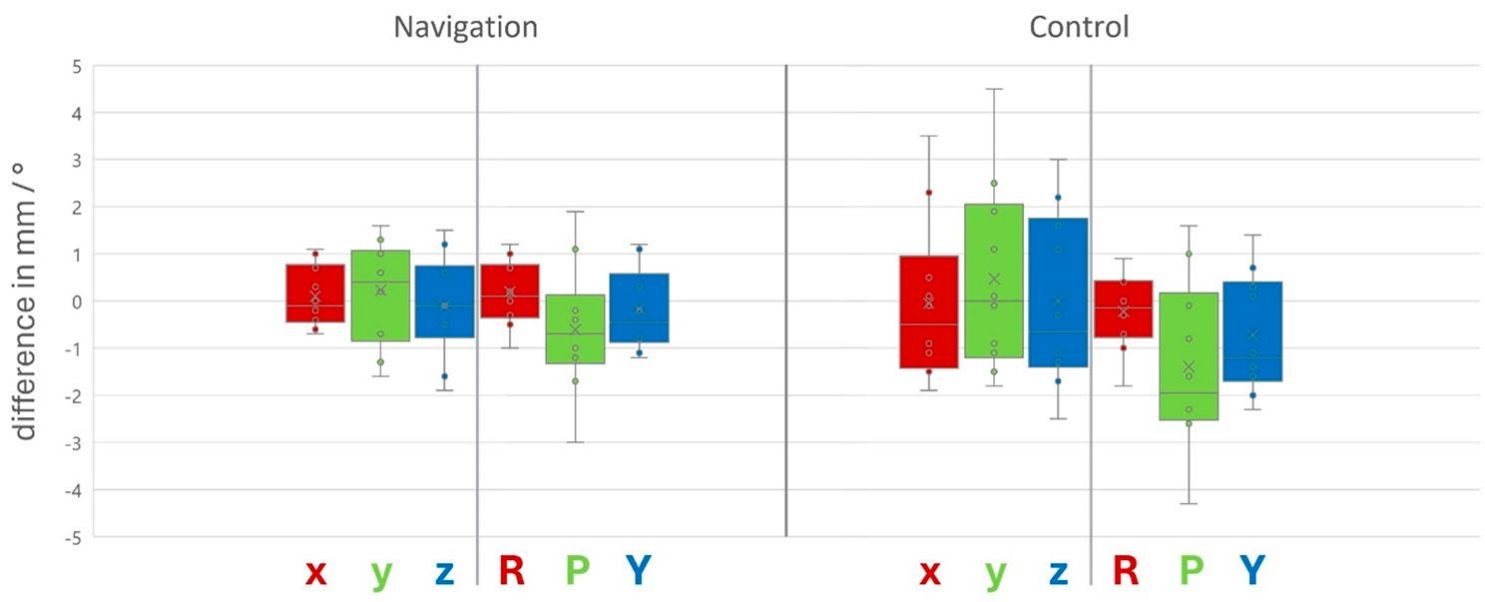
Boxplots depicting data distribution for the difference between postoperatively achieved and pre-operatively planned positioning of the maxilla for both groups. Data is shown for each axis along the translational movements in millimeter (x: right-left, y: back-front, z: down-up) and rotational movements in degree (Roll, Pitch and Yaw)

Figure 6 presents boxplots showing the distribution of differences between post-surgical and planned maxillary positioning across all three spatial axes for translational and rotational movements in both the experimental and control groups.

To determine whether navigation improved surgical accuracy, t-tests were conducted on the absolute values of the measurements, disregarding overcorrection or undercorrection to prevent misleading conclusions from symmetrically distributed data around zero. Shapiro-Wilk tests confirmed normal distribution of the data, while variance homogeneity testing using Levene‘s tests and Brown-Forsythe tests showed equal variance distribution across all movements except for translational movement along x-axis (see supplementary Table 1 in supplementary material). Therefore, Welch’s t-test was used for this movement, while Student’s t-test was used for all other movements. The results, shown in Table 2, indicate that navigation led to more precise outcomes compared to the non-navigated control group across all movements. Significant improvements were observed in translational movements along the x-axis (right-left) and z-axis (down-up), as well as in yaw rotation, with surgical accuracy improved by -0.81 mm (*p* = 0.021), -0.82 mm (*p* = 0.014), and − 0.45° (*p* = 0.045), respectively. Other movements also showed more precise results in the navigated group, though not reaching statistical significance: -0.60 mm (*p* = 0.094) along the y-axis (back-front), -0.70° (*p* = 0.071) in pitch rotation, and − 0.04° (*p* = 0.428) in roll rotation.

**Table 2:** Comparison of surgical accuracy between experimental group (navigated) and control group (non-navigated)

Student’s t-test	Translation			Rotation		
	x (right-left)	y (back-front)	z (down-up)	Roll	Pitch	Yaw
variances	unequal	equal	equal	equal	equal	equal
mean difference	**-0.81**	**-0.60**	**-0.82**	**-0.04**	**-0.70**	**-0.45**
SE of the difference	0.35	0.44	0.34	0.22	0.46	0.25
lower 95% CI	-1.58	-1.52	-1.54	-0.49	-1.66	-0.98
upper 95% CI	-0.04	0.32	-0.10	0.41	0.26	0.08
t	-2.30	-1.37	-2.40	-0.19	-1.54	-1.79
df	11.1	18	18	18	18	18
p	**0.021**	0.094	**0.014**	0.428	0.071	**0.045**
Cohens d	1.03	0.61	1.07	0.08	0.69	0.80
Power	0.72	0.37	0.74	0.07	0.44	0.53

Negative value for the mean difference shows that the surgical accuracy improves for navigation as mean deviation of the achieved maxillary position from the planned position decreases by the shown value in mm for translation and ° for rotation compared to the control group. T-tests were calculated (Student’s for equal and Welch’s for unequal variance). *P*-values of *p* < 0.05 are considered statistically significant and are highlighted in bold text

SE: standard error; CI: confidence interval; t: t-statistic; df: degree of freedom; *p*: *p*-value; Cohens d: measure of effect size; Power: calculated power of post-hoc power analysis

**Fig. 7:**
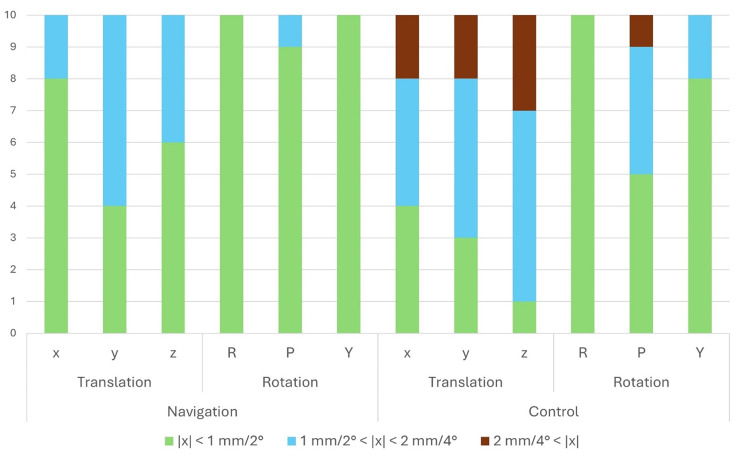
Distribution to Target intervals. Achieved accuracies for both experimental and control groups are assigned to target intervals according to clinically accepted surgical accuracies: “good” (in green color) for values less than 1 mm for translational movements (x-, y- and z-axis) and less than 2° for rotational movements (roll, pitch and yaw rotation), “acceptable” (in blue color) for values between 1 mm/2° and 2 mm/4°, and “undesirable” (in red color) for values exceeding 2 mm/4°

To evaluate the clinical significance of the increased surgical accuracy, values were categorized into target intervals based on accepted clinical standards: less than 2 mm for translational movements and less than 4° for rotational movements. Intervals were defined as “good” for values under 1 mm/2°, “acceptable” for values between 1 mm/2° and 2 mm/4°, and “undesirable” for values exceeding 2 mm/4°. Figure 7 illustrates the distribution of absolute mean values to the defined target intervals for both groups. In the navigated group, no values (0%; 0/60) were “undesirable,” while in the control group, 13.3% (8/60) exceeded the accepted threshold. These inaccuracies were observed across all translational movements and in the pitch rotation. Overall, 78.3% (47/60) of the values in the experimental group fell within the “good” interval, compared to 50% (30/60) in the control group.

A mixed ANOVA was conducted to identify factors influencing surgical accuracy (see Table 3). Surgical accuracy, the dependent variable, was calculated as the root mean square of the difference between the planned and achieved maxillary positions across all translational and rotational movements. The factors analyzed for their impact on surgical accuracy included the A) use of surgical navigation, B-D) discrepancies along the three spatial axes (sagittal, as Angle Class; vertical, as the presence of open bite; and lateral, as facial scoliosis), and E) the planned overall surgical movement of the maxilla, measured as the root mean square of the difference between the pre-operative and planned positions across all movements. The planned movement was categorized into the two categories ≤ 2 and > 2.

Shapiro-Wilk tests confirmed normal distribution of the data, while variance homogeneity testing using Levene‘s tests and Brown-Forsythe tests showed equal variance distribution across all groups (see supplementary Table 2 in supplementary material). Usage of surgical navigation and the amount of planned movement significantly influenced surgical accuracy, with *p* = 0.004, η²_p_=0.45, and *p* = 0.037, η²_p_=0.24, respectively. Other factors had no significant impact. To determine whether specific orthognathic cases particularly benefit from surgical navigation, interactions between the factor A usage of surgical navigation and the other factors B-E were analyzed (see Table 3). No significant interactions were found, thus no factor among those examined could be identified as a predictor for cases that would particularly benefit from usage of surgical navigation.

**Table 3:** Mixed ANOVA for analysis of influencing factors on surgical accuracy calculated as the root mean square of the difference between planned and post-surgically acquired maxillary position across all translational and rotational movements

Mixed ANOVA		sum of squares of type III	df	mean of squares	F	*p*	η^2^_*p*_	Power
A	surgical navigation	2.31	1	2.31	11.56	**0.004**	0.45	0.97
B	sagittal discrepancy (Angle Class)	0.47	2	0.23	1.17	0.340	0.14	0.29
C	vertical discrepancy (Open Bite)	0.07	1	0.07	0.32	0.582	0.02	0.09
D	lateral discrepancy (Face Asymmetry)	0.41	1	0.41	2.28	0.150	0.12	0.35
E	planned movement (pre-op to planning)	0.74	1	0.74	5.18	**0.037**	0.24	0.66
A x B	navigation x sagittal discrepancy	0.17	2	0.09	0.43	0.657	0.06	0.14
A x C	navigation x vertical discrepancy	0.06	1	0.06	0.27	0.609	0.02	0.09
A x D	navigation x lateral discrepancy	0.18	1	0.18	0.99	0.334	0.06	0.19
A x E	navigation x amount of movement	0.40	1	0.40	2.76	0.116	0.15	0.43

*p*-values of *p* < 0.05 are considered statistically significant and are highlighted in bold text

df: degree of freedom; F: F-statistic; *p*: *p*-value, η²_p_: partial eta-squared; RMS: root mean square; Power: calculated power of post-hoc power analysis

## Discussion

This study presents and evaluates a comprehensive protocol for usage of optical navigation to enhance splint-based maxillary positioning control during orthognathic surgery. Significant improvements in surgical accuracy were observed, particularly in horizontal (left-right), vertical (up-down), and yaw rotational movements. While roll rotation showed minimal improvement, enhancements in pitch rotation and sagittal (forward-backward) movements were noted.

The utilization of navigation in orthognathic surgery has not been well-established but documented in several studies, primarily focusing on anatomical orientation to enhance surgical security and facilitating plan transfer [26]. While this technology proved useful for indicating osteotomy lines, positioning the ramus and controlling maxillary positioning, protocols described vary significantly across reported studies making it challenging to establish consistent practices [26]. The present protocol distinguishes itself with two key features: (1) a non-invasive registration method, which avoids the need for surgically placed opaque markers or radiation-based imaging, thereby eliminating additional invasiveness or data acquisition; and (2) a point-based evaluation method integrated into the intermediate splint design, enabling precise and reproducible intraoperative reassessment of the planned maxillary position.

The image-to-patient registration process is a critical determinant of surgical accuracy in navigation systems. While various established approaches exist — such as point-based (marker- or anatomy-driven), surface-based, and computer-vision-based registration methods — each entails a trade-off between accuracy, robustness, invasiveness, and time efficiency [31]. Given the high accuracy requirements in orthognathic surgery, most protocols rely on point-based registration methods using hard tissue-supported landmarks [26]. These include anatomical structures, such as bony or dental landmarks, which may lack defined geometries and thereby limit accuracy in certain patients, or invasive, bone- or dental-anchored markers. Zinser et al. report that even with usage of geometrically well-defined landmarks like orthodontic brackets, image-to-patient registration lack accuracies because of metallic scatter artifacts [23]. Similar problems in identifying opaque markers in the virtual data set have been reported by Eckstein et al. who investigated fiducial registration errors of dental vacuum splints limiting overall surgical accuracy. Thus, a precise identification of opaque markers in the DICOM data set as a target to be reached can be difficult and may even lead to less accuracy than compared to dental or anatomical landmarks [32]. The presented protocol addresses these challenges by integrating registration landmarks directly into the virtual occlusal splint. This eliminates the need for additional planning data and further radiological imaging posing radiation exposure to the patient while offering the same high accuracy as derived from bone-anchored fiducial marker-based registration [27]. This is achieved through the matching algorithm of the orthognathic planning software, which aligns high-resolution intraoral scans with the DICOM data to which the patient must be registered [33]. Due to the precise matching and the excellent fit of the splints, this non-invasive registration protocol has demonstrated registration accuracy during validation in other contexts for both optical and electromagnetic tracking [27, 34, 35]. Optical tracking is favored for its higher accuracy compared to electromagnetic tracking, though it requires a direct line of sight between the camera and the tracked object [36]. In craniofacial surgery, the use of bone-fixed skull reference arrays is common, providing robust tracking but requiring invasive fixation to the skull [37]. However, a small incision behind the hairline during the orthognathic procedure represents relatively minimal invasiveness compared to the procedure itself. Alternatives like headbands are less invasive but offer reduced accuracy due to potential slippage [38]. However, Shirota et al. have reported good outcomes with headbands for usage of navigation in orthognathic surgery, though they emphasized that the registration process becomes even more critical, as slippage of the headband may necessitate repeated re-registrations [28].

The practical implementation of the intraoperative evaluation of maxillary positioning via navigation is rarely detailed in literature. Typically for pointer-based navigation with optical tracking, it involves intra-operative pointing on surfaces or landmarks and screen monitoring if the pointed surface/landmark matches the planned STL surface or defined landmark [39–42]. However, this method is especially complex for 3D objects due to their complex geometry and the fact that point-based evaluation only allows control of one coordinate at a time. This can lead to mismatches even if some pointed surfaces appear congruent. If anatomical landmarks with a more recognizable geometry are used for evaluation like the tips of canines or incisors, these can be unreliable due to tooth abrasion and the technical difficulty of pinpointing exact locations on hard and smooth enamel surfaces. If an offset is identified between the actual position and the virtually planned position, there is no established best practice for precisely evaluating the spatial deviation to guide the necessary correction of the position [28, 43]Sun et al. utilized defined offsets of the navigation probe to estimate the required movement along different axes based on the probe’s angulation during evaluation [44]. However, this method is susceptible to errors due to the swinging of the navigation probe, which can amplify the navigation system’s inaccuracies. Berger et al. introduced a system based on electromagnetic tracking which allowed the real-time display of the maxilla as a 3D-object along with its current deviation across the three spatial axes for both translational and rotational movements. They reported promising results for navigated free-hand positioning of the maxilla in a pilot study, demonstrating surgical accuracy comparable to the splint-based approach, while noting the increased difficulty of the technically demanding free-hand positioning [45–47]. The possibility to track the maxilla as a 3D object with real-time information of translational and rotational movements along al three spatial axes has to be seen as an advantage over the point-based method being utilized with optical navigation. However, a drawback of electromagnetic tracking is the increased demands on the operating room and instrumentation to ensure compatibility, which if lacking may lead to accuracy issues in the presence of electromagnetic interference [36].

The presented protocol involves an optical navigation system and addresses the above-mentioned issues by defining virtual 3D coordinates within the indentations of the intermediate splint that are easily identifiable and reproducibly navigable intraoperatively, ensuring precise transfer from virtual to real-world settings and enabling a true point-to-point comparison. The high accuracy of this process is ensured through the integration and correct alignment of the high-resolution intraoral scan with the DICOM dataset, which also results in the excellent fit of the 3D-printed splints [33]. Alternative methods, such as trajectory-based evaluation used in orbital implant placement [16], were also tested in pilot cases of this study but were found unsuitable due to the specific requirements of maxillary positioning. In maxillary repositioning combined with splints, the primary objective is to verify the accuracy of the planned movement while identifying areas needing further bone trimming. Trajectory-based evaluation is less effective in this context because it does not accommodate the simultaneous bone trimming necessary for maxillary adjustments. The point-based approach, which assesses discrepancies at specific reference points (paranasal and zygomaticoalveolar crest), aligns more closely with traditional model surgery practices. Combining surgical navigation with a splint-based approach avoids the technical challenges of free-hand positioning with navigation alone [42, 47], and leverages the strengths of both methods. This integration allows for simpler osteosynthesis with stable jaw fixation via the splint, while also enabling intraoperative position control referenced to the skull base, rather than the mobile mandible. Using skull-referenced navigation mitigates errors such as inaccurate bite registration that could distort the condyle-fossa relationship during CBCT scanning and lead to occlusal splint inaccuracies [48]. Thus, this approach potentially offers greater accuracy over the splint-only approach as supported by the results of the present study.

The presented method provides a transparent and precise approach, making navigation in orthognathic surgery more feasible and comprehensible. One key advantage is the flexibility to decide on-the-fly whether to use navigation, without the extensive preparation required for PSIs (patient-specific implants). This method integrates smoothly into existing CAD/CAM splint workflows and can be combined with minimally invasive techniques, allowing skull-referenced plan transfer without PSI’s limitations. Although the results are promising, the study did not assess the clinical impact of the observed improvement in surgical accuracy. Long-term studies are needed to determine whether increased surgical accuracy leads to tangible clinical benefits, such as improved long-term stability, reduced recurrence rates, enhanced quality of life or higher patient satisfaction. Similar evidence is also lacking for PSIs [11].

One of the main limitations of this study is the small sample size. Although comparable studies included similarly small patient cohorts, the statistical significance of the results is limited, as evidenced by the relatively low power indicated in the post-hoc power analysis. Therefore, this study should be considered more of a pilot study, providing promising preliminary findings. However, future studies should include a larger patient cohort to establish stronger statistical significance between the navigation and control groups, with enhanced power and confidence in the results.

A key strength of the study is that it is a case-control design with an appropriate control group, which enhances the reliability of the results. Blinding was only possible for the evaluator following pseudonymization, which is inherent to the nature of the surgical procedure. Another strength is the use of state-of-the-art evaluation methods, such as voxel-based matching, thus eliminating discrepancies from multiple landmark identifications and recognition errors between various observer. This voxel-based evaluation, considered the most accurate method [49], is now integrated into some orthognathic planning software and should be the standard for assessing surgical accuracy in orthognathic surgery [50]. The reporting of deviations in maxillary positioning also follows the current standard, with translational and rotational movements of the maxilla along all three spatial axes.

Despite all discussed advantages, surgical navigation has its limitations. Accuracy can be compromised if any part of the workflow deviates from high standards. Navigation is more of a verification tool than a positioning tool, the surgeon’s expertise is still critical for accurate positioning, with navigation serving to confirm success. Moreover, a certain expertise in utilizing navigation has to be build with this protocol. In particular, there were 5 pilot cases for testing and refining the initial protocol to further improve technical details. Navigation initially extends surgery duration, although we observed a learning curve which led to a decrease of the additional time with experience as the team became more proficient. Repeated use of navigation also enhances overall system proficiency, benefiting other surgical applications of navigation and offering significant advantages in training young surgeons. Furthermore, the cost-effectiveness of the navigation-based approach in orthognathic surgery may vary significantly depending on the existing resources at a facility. While potential cost savings are noted, the need for specialized hardware, such as the Brainlab system, can introduce substantial costs, particularly for institutions that do not already have this equipment. These costs include the initial investment in the system, setup, maintenance, and necessary training. Additionally, the time required for system setup and navigation during surgery can increase operating room costs, potentially reducing the overall cost-effectiveness compared to PSIs. On the other hand, it should be considered that the navigation system can be used for a variety of other indications and can even be shared with other disciplines, which helps to put the high acquisition costs into perspective. Furthermore, cost savings should also consider long-term benefits, such as improved surgical outcomes and reduced post-operative complications, which may lead to downstream savings. A comparable study on the use of navigation in knee arthroplasty identified a breakeven point at $629, where cost savings enabled by navigation were achieved, provided that the costs of navigation per procedure remained below this threshold [51]. Comprehensive cost analyses for usage in orthognathic surgery, including both short-term and long-term factors, is difficult to assess due to the limited data and should be further elucidated in future studies to fully assess the financial viability of this approach.

While PSI remains highly accurate and time-efficient, especially in complex cases like multi-segmental procedures or those involving cleft patients with poor bone quality [52], we see a future for surgical navigation in orthognathic surgery. As new technologies like mixed reality with real-time alignment for in-situ visualization emerge, which rely on similar principles as conventional navigation, there is a growing need for non-invasive yet accurate registration and intraoperative evaluation methods [22]. The presented and evaluated workflow addresses this need, making it valuable for future developments in conventional and mixed reality-driven navigation processes.

## Conclusion

This study clinically demonstrated that CAD/CAM splints, generated by aligning DICOM data of the facial skeleton with STL data from intraoral scans, provide precise registration for surgical navigation in orthognathic patients without the need for additional imaging or invasive fiducials. The modification of the intermediate splint enabled reliable intraoperative control of the maxillary position, proving this method to be highly applicable in clinical practice. The results support enhanced surgical accuracy, favoring the use of navigation according to the presented protocol.

In conclusion, navigation offers significant value in orthognathic surgery when accurate, non-invasive registration methods and a standardized real-time evaluation protocol are used. This approach improves surgical accuracy in established workflows, such as the CAD/CAM splint method, without requiring additional invasive imaging or registration steps, making it practical and efficient for routine clinical application.

## Electronic supplementary material

Below is the link to the electronic supplementary material.

Supplementary Material 1

## Data Availability

Data is provided within the manuscript.
