# Supplementary material for: Novel CAD/CAM-splint-based navigation protocol enhances intraoperative maxillary position control in orthognathic surgery: a case control study
Source: Head Face Med. 2025 Jan 10;21:1. doi: 10.1186/s13005-024-00477-3 (PMC11721267; doi:10.1186/s13005-024-00477-3)
Supplement: Supplementary file 1 — Supplementary Material 1 [file 13005_2024_477_MOESM1_ESM.docx]

**Supplementary material**

**Supplementary table 1: Assumptions for t-tests**

Test for normality and variance homogeneity testing was performed for the data of absolute mean values of the difference between planned and postoperative position for both groups across all movements across all axes. P-values of p < 0.05 are considered statistically significant.

W: value of W-statistics; p: p-value; F: F-statistic; df: degree of freedom

| **Assumptions**  **for t-tests** | | | *Translation* | | | *Rotation* | | |
| --- | --- | --- | --- | --- | --- | --- | --- | --- |
|  |  |  | **x**  **(right-left)** | **y**  **(back-front)** | **z**  **(down-up)** | **Roll** | **Pitch** | **Yaw** |
| Normality | Shapiro-Wilk Test | **W** | 0.95 | 0.92 | 0.87 | 0.93 | 0.92 | 0.90 |
|  |  | **p** | 0.702 | 0.357 | 0.090 | 0.479 | 0.344 | 0.212 |
| Variance homogeneity | Levene's Test | **F** | 5.41 | 2.82 | 0 | 0.11 | 1.09 | 2.52 |
|  |  | **df1** | 1 | 1 | 1 | 1 | 1 | 1 |
|  |  | **df2** | 18 | 18 | 18 | 18 | 18 | 18 |
|  |  | **p** | 0.032 | 0.111 | 0.945 | 0.744 | 0.310 | 0.130 |
|  | Brown-Forsythe Test | **F** | 5.32 | 2.50 | 0.01 | 0.02 | 1.11 | 1.83 |
|  |  | **df1** | 1 | 1 | 1 | 1 | 1 | 1 |
|  |  | **df2** | 18 | 18 | 18 | 18 | 18 | 18 |
|  |  | **p** | 0.033 | 0.131 | 0.922 | 0.886 | 0.307 | 0.193 |

**Supplementary table 2: Assumptions for ANOVA**

Test for normality and variance homogeneity testing was performed for the data of the residuals of each group of the ANOVA. P-values of p < 0.05 are considered statistically significant.

W: value of W-statistics; p: p-value; F: F-statistic; df: degree of freedom; Y: yes; N: no

| **Assumptions**  **for ANOVA** | | | | *Angle Class* | | | *Open Bite* | | *Face Asymmetry* | | *Planned movement* | |
| --- | --- | --- | --- | --- | --- | --- | --- | --- | --- | --- | --- | --- |
|  |  |  |  | **I** | **II** | **III** | **Y** | **N** | **Y** | **N** | **≤2** | **>2** |
| Normality | Shapiro-Wilk | *N* | **W** | 0.95 | 0.92 | 0.87 | 0.94 | 0.94 | 0.87 | 0.93 | 0.85 | 0.92 |
|  |  |  | **p** | 0.604 | 0.323 | 0.272 | 0.650 | 0.648 | 0.272 | 0.617 | 0.240 | 0.530 |
|  |  | *C* | **W** | 0.95 | 0.84 | 0.74 | 0.92 | 0.79 | 0.90 | 0.79 | 0.78 | 0.85 |
|  |  |  | **p** | 0.604 | 0.209 | 0.059 | 0.538 | 0.053 | 0.382 | 0.086 | 0.053 | 0.189 |
| Variance homogeneity | Levene's Test |  | **F** | 1.46 | | | 1.31 | | 0.80 | | 0.78 | |
|  |  |  | **df1** | 5 | | | 3 | | 3 | | 3 | |
|  |  |  | **df2** | 14 | | | 16 | | 16 | | 16 | |
|  |  |  | **p** | 0.264 | | | 0.305 | | 0.514 | | 0.520 | |
|  | Brown-Forsythe Test |  | **F** | 0.62 | | | 0.38 | | 0.76 | | 0.47 | |
|  |  |  | **df1** | 5 | | | 3 | | 3 | | 3 | |
|  |  |  | **df2** | 14 | | | 16 | | 16 | | 16 | |
|  |  |  | **p** | 0.689 | | | 0.769 | | 0.531 | | 0.710 | |
